# Effects of High-Intensity Interval Training on Melatonin Function and Cellular Lymphocyte Apoptosis in Sedentary Middle-Aged Men

**DOI:** 10.3390/medicina59071201

**Published:** 2023-06-26

**Authors:** Hadeel A. Al-Rawaf, Sami A. Gabr, Amir Iqbal, Ahmad H. Alghadir

**Affiliations:** 1 Department of Clinical Laboratory Sciences, College of Applied Medical Sciences, King Saud University, Riyadh 11433, Saudi Arabia; hadeelar12345@gmail.com; 2 Department of Rehabilitation Sciences, College of Applied Medical Sciences, King Saud University, Riyadh 11433, Saudi Arabia; dr.samigabr@gmail.com (S.A.G.); aalghadir@hotmail.com (A.H.A.)

**Keywords:** melatonin, lymphocyte apoptosis, HIIT training, cytochrome c oxidase

## Abstract

*Background*: Physical performance increased by controlled interventions of high-intensity intermittent training (HIIT); however, little is known about their influence as anti-aging and antioxidant effects, or their role in mitochondrial biogenesis. *Purpose*: This study aimed to determine the effects of HIIT for 12 weeks on melatonin function, lymphocyte cell apoptosis, oxidative stress on aging, and physical performance. *Methods*: Eighty healthy male subjects aged 18–65 years randomly participated in a HIIT-exercise training program for 12 weeks. Anthropometric analysis, cardiovascular fitness, total antioxidant capacity (TAC), lymphocyte count and apoptosis, and serum melatonin and cytochrome c oxidase (COX), were estimated for all subjects before and after HIIT-exercise training. HIIT training was performed in subjects for 12 weeks. *Results*: Data analysis showed a significant increase in the expression levels of the melatonin hormone (11.2 ± 2.3, *p* < 0.001), TAC (48.7 ± 7.1, *p* < 0.002), COX (3.7 ± 0.75, *p* < 0.001), and a higher percentage of lymphocyte apoptosis (5.2 ± 0.31, *p* < 0.003). In addition, there was an improvement in fitness scores (W; 196.5 ± 4.6, VO_2_max; 58.9 ± 2.5, *p* < 0.001), adiposity markers (*p* < 0.001); BMI, WHtR, and glycemic control parameters (*p* < 0.01); FG, HbA1c (%), FI, and serum C-peptide were significantly improved following HIIT intervention. Both melatonin and lymphocyte apoptosis significantly correlated with the studied parameters, especially TAC and COX. Furthermore, the correlation of lymphocyte apoptosis with longer exercise duration was significantly associated with increased serum melatonin following exercise training. This association supports the mechanistic role of melatonin in promoting lymphocyte apoptosis either via the extrinsic mediator pathway or via inhibition of lymphocyte division in the thymus and lymph nodes. Additionally, the correlation between melatonin, lymphocyte apoptosis, TAC, and COX activities significantly supports their role in enhancing physical performance. *Conclusions*: The main findings of this study were that HIIT exercise training for 12 weeks significantly improved adiposity markers, glycemic control parameters, and physical performance of sedentary older adult men. In addition, melatonin secretion, % of lymphocyte apoptosis, COX activities, and TAC as biological aging markers were significantly increased following HIIT exercise training interventions for 12 weeks. The use of HIIT exercise was effective in improving biological aging, which is adequate for supporting chronological age, especially regarding aging problems. However, subsequent studies are required with long-term follow-up to consider HIIT as a modulator for several cardiometabolic health problems in older individuals with obesity.

## 1. Introduction

Human aging is experienced with increased incidences of chronic and serious diseases. Higher rates of heart disease, diabetes, cancer, and declining cognitive capacities such as reduced strength, impaired hearing, and worse memory were reported at the same rate among all individuals with identical chronological ages [1,2]. 

However, a remarkable variation in the rate of biological aging was identified among individuals with identical chronological ages. This, in turn, might be associated with a variation in the incidence of faster age-related declines in health among some adults than others [3,4].

Biological aging refers, in turn, to the gradual and progressive decline in the physiological functions simultaneously involving multiple organ systems within human bodies [5]. Thus, the consequences of differences in genetic endowment, cellular biology, and cellular physiological changes with the accumulation of life experiences across one’s lifespan lead to the divergence of biological age from chronological age among some individuals [6,7,8,9]. 

In addition, acceleration in biological aging is measured by physiological and cellular changes among older adults of the same chronological age. These adults are likely to develop serious diseases such as heart disease, diabetes, and cancer, and have a higher rate of cognitive decline, disability, and mortality [10,11,12].

In midlife, chronological age is adequate to support aging problems, particularly among individuals with rapid aging than other same-age peers [13,14,15]. Thus, the need to study and explore biological aging among older adults or midlife people is required to support and explain aging-related changes [13,14,15].

Biological aging biomarkers have been previously developed and validated along with phenotypic aging as considerable and important markers for identifying the changes that accompany human aging [16,17].

Indeed, understanding the cellular apoptosis of the most important biological systems, such as the immune system and other hormonal changes, as biological aging biomarkers, was required among older adults. Such a way provides more information to build up future models of biological aging.

Apoptosis or cell death was shown as the most important biological mechanism for regulating immune response [18,19]. The killing and elimination of the pathogens needs to induce cell death via apoptosis to activate immune cells, whereas apoptosis acts as a regulatory process to terminate the immunological response [20,21]. Thus, abnormal apoptosis or a missed off-switch regarding apoptosis was significantly reported in chronic inflammation or self-reactive immune responses.

Melatonin (N-acetyl-5 methoxy tryptamine) is a hormone secreted from the pineal gland according to a circadian pattern [22]. Previous studies showed that melatonin could play a role in modulating various biological functions with anti-aging effects [23], anti-inflammatory effects [24], and anti-oxidant activities [25,26]. Furthermore, it protects against chronic diseases, such as cardiovascular diseases, diabetes, and obesity [27,28,29]. Additionally, physically active bodies and practicing exercise are the most significant agents that affect biological aging and protect human bodies from serious diseases among older adults [30,31,32,33].

Physical exercise is very important for maintaining healthy bodies. Physical fitness—including healthy weights, bones, muscles, and joints—physiologically promotes the body against falls and weakness, and reduces surgical risks [34]. Exercise with various intensities and duration was shown to play a role in strengthening the immune system [35]. It was reported that exercise exerts significant hormonal changes, which may affect the mobilization of lymphocytes. Exercise of moderate types was previously shown to increase the secretion of endocrine hormones, lower accumulation of autoreactive immune cells, and subsequently enhance programmed cell death (apoptosis), which reverses the senescence of immune cells. Thus, exercise through physiological hormonal mechanisms can reverse the senescence of immune cells [36].

PA was shown to reduce susceptibility to cancer or degeneration of human cells via increasing melatonin hormonal levels, reducing estrogen production, and improving fat metabolism [37,38]. Additionally, positive apoptotic regulation was observed in various human cell types following participation in moderate exercise training by modulating several physiological factors such as DNA damage, cytokine, reactive oxygen species (ROS), and hormone levels [39].

In metabolically active tissues, the physical activity resulting from varying exercise modes is considered one of the best-known strategies to improve physical performance and overall human health [40,41].

High-intensity interval training (HIIT) as a mode exercise program led to higher improvements than moderate-intensity continuous training (MICT) on functional capacities, muscle function, body composition, and blood biomarkers in obese older adults [42]. In addition, it improves lean mass and skeletal muscle markers of mitochondrial content, fusion, and mitophagy [42].

Thus, as a time-efficient type of training, HIIT could be recommended as an exercise modality to maintain or improve mobility, health, and quality of life among older adults with obesity and other diseases [42,43]. Globally, high-intensity interval training (HIIT) as one type of the traditional exercise modes and other personal and functional fitness training modes scored relatively highly applicable in Europe compared with the United States [44].

However, little is known about the effects of high-intensity interval training (HIIT) on apoptosis regulation mechanism, melatonin function in aging, physical performance, and mitochondrial biogenesis. Thus, in this current study, the effects of melatonin secretion, lymphocyte cell apoptosis, and oxidative stress on aging and physical performance were evaluated in healthy male subjects following HIIT training for 12 weeks.

## 2. Materials and Methods

### 2.1. Subjects

**Figure 1:**
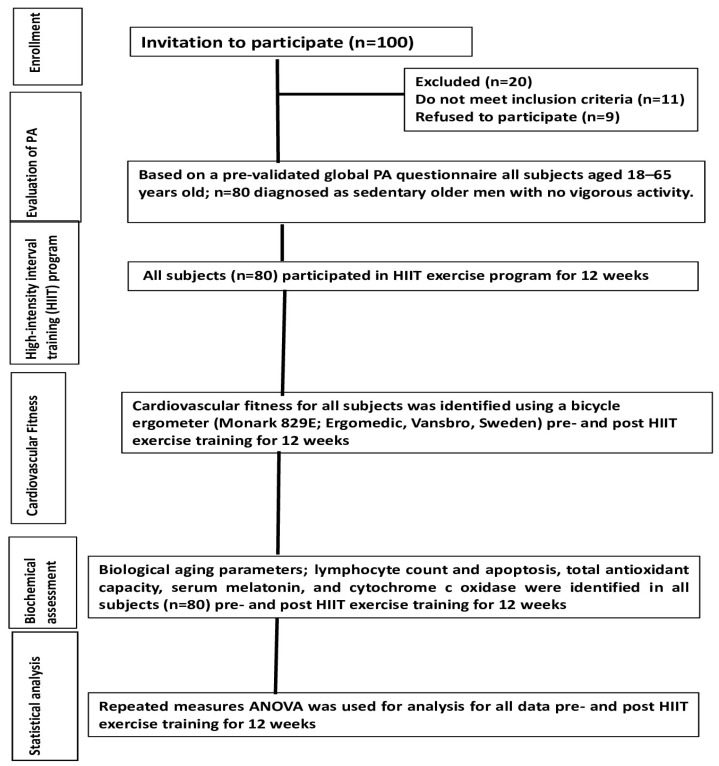
Outline of study procedures.

A total of 100 healthy male subjects aged 18–65 years were randomly nominated for this study after having gave written, informed consent. Only 80 subjects who matched the inclusion criteria participated in this study (Table 1 and Figure 1). As the study was performed on healthy subjects, there was a lack of a control group. Instead, the potential effects of HIIT exercise on biological and adiposity markers were estimated from pre-test baseline data and post-HIIT intervention data for 12 weeks. None of the participated subjects had a history of metabolic bone or eating disorders, or chronic or infectious diseases. Additionally, subjects with physical disabilities and who had drugs that could interfere with endocrine hormone secretion, immunity, and antioxidant capacity were excluded from this study. All participants of this study were instructed to have their normal diet during data collection. Anthropometric and body adiposity markers of interest such as body mass index (BMI), hips, waist-to-height ratio (WHtR), conicity index (C-index), and body adiposity index (BAI) were measured using standard measurements before and after training sessions as previously reported [21,22]. This study was approved by the ethical committee of the Ethics Sub-Committee of King Saud University under file number ID: RRC-2017-068.

### 2.2. Exercise Training Program

All subjects were sedentary males identified using a pre-validated global PA questionnaire as previously reported [45,46,47,48]. In that questionnaire, subjects with low activity refers to those with a sedentary lifestyle who have no PA during work and transportation. All subjects participated in a high-intensity interval training (HIIT) program for 40 min/3 sessions/week for 12 weeks using an electronic treadmill (Vegamax, made in Taiwan). The entire HIIT program was performed in the RRC lab, CAMS, King Saud University, and was supervised by an expert physiotherapist with >10 years of experience in his respective field.

The HIIT program consisted of 4 × 4 min intervals at 80–85% of HRmax, with 3 min active recovery at 70% of HRmax between intervals. All subjects started a warm-up for 10 min at 50% of maximal heart rate (HRmax) and went through a 5 min cooldown before initial HIIT program sessions, as previously reported [23]. The heart rate, Borg scale of perceived exertion, as well as the adjustment of the speed of the treadmill were monitored during training to ensure that all subjects were exercising at their corresponding intensity of exercise and to avoid training adaptations during the whole period of the training program. Before and after exercise, venous blood samples (5 mL) were collected from all subjects to estimate melatonin, cytochrome c, lymphocyte count, and apoptosis.

### 2.3. Assessment of Cardiovascular Fitness

A bicycle ergometer (Monark 829E; Ergomedic, Vansbro, Sweden) was used to measure cardiovascular fitness in all subjects. Every third minute, the bicycle was programmed to increase the workload by 50 watts for men and 40 watts for females until exhaustion. A polar heart rate monitor fixed around the chest measured heart rate. In addition, a subjective assessment by a trained observer was performed to determine the exhaustion state, which refers to the participant’s inability to continue training, even with encouragement. To calculate the maximal power output (in Watts [W]), the following formula was used [24], [{W1 + (W2 − W1) × *T/180]}, in which W1 (W) corresponds to workload at the last fully completed stage, W2 (W) corresponds to workload at the final incomplete stage, and T (s) corresponds to time at the last incomplete stage. Cardiovascular fitness was expressed as the maximal output (W) [24].

### 2.4. Assessment of Lymphocyte Count and Apoptosis

Lymphocyte cells were separated from peripheral blood samples (2 mL) to estimate lymphocyte count and viability using trypan blue exclusion and separation tests, as reported previously [25,26,27]. In addition, the DNA-labeling technique was performed to assess lymphocyte apoptosis, which uses acridine orange as a binding fluorescent dye, as mentioned previously in the literature [28].

### 2.5. Assessment of Total Antioxidant Capacity (TAC)

Serum samples of all participants were tested to estimate antioxidant capacity (TAC) as previously reported in the literature [29,30,31]. A colorimetric assay kit (Catalog #K274-100; BioVision Incorporated, CA 95035, USA) was used to perform TAC in this test. The equivalent concentrations of TAC were measured colorimetrically at 570 nm as a function of Trolox concentrations according to the manufacturer’s instructions [29,30,31]. The results reported are calculated according to the following formula;

{[Sa/Sv = nmol/µL or mM Trolox equivalent

where Sa is the sample amount (in nmol) read from the standard curve, and Sv is the undiluted sample volume added to the wells) [29,30,31].

### 2.6. Assessment of Serum Melatonin and Cytochrome C OxidaseX

Immune assay ELISA kits (RE54021. IBL Gesellschaft fur Immunochemie und Immunbiologie MBH, Flughafenstrasse, Hamburg, Germany) were used to estimate serum melatonin in all subjects before and after exercise training. Furthermore, cytochrome c oxidase (COX) was measured in serum samples with competitive ELISA kits obtained from Chemicon International, Temecula, CA, USA. The cytochrome c ELISA kit procedures were performed according to the manufacturer’s instructions [32].

### 2.7. Statistical Analysis

Statistical analysis was performed using SPSS software, version 20.0 (SPSS Inc., Chicago, IL, USA), and results were expressed as mean ± standard deviation (SD). Repeated measures ANOVA was performed to compare changes in the pre- and post-training mean values of BMI, WHtR, melatonin, cytochrome c, TAC, and lymphocyte apoptosis. In addition, the association between serum melatonin and lymphocyte apoptosis (%) with other variables—such as adiposity markers, fitness, TAC, and cytochrome C—were calculated using correlation coefficients (r and β) as tests adjusted for age (42.5 ± 3.1). All data levels obtained at *p* < 0.05 were reported as significant.

## 3. Results

Eighty out of a hundred healthy male subjects aged 18–65 years were recruited in this study. Demographic and clinical characteristics are shown in Table 1. To study the influence of HIIT exercise on the expression levels of melatonin, antioxidant capacity, and related apoptotic markers, the subjects participated in a supervised HIIT exercise program for 12 weeks.

**Figure 2:**
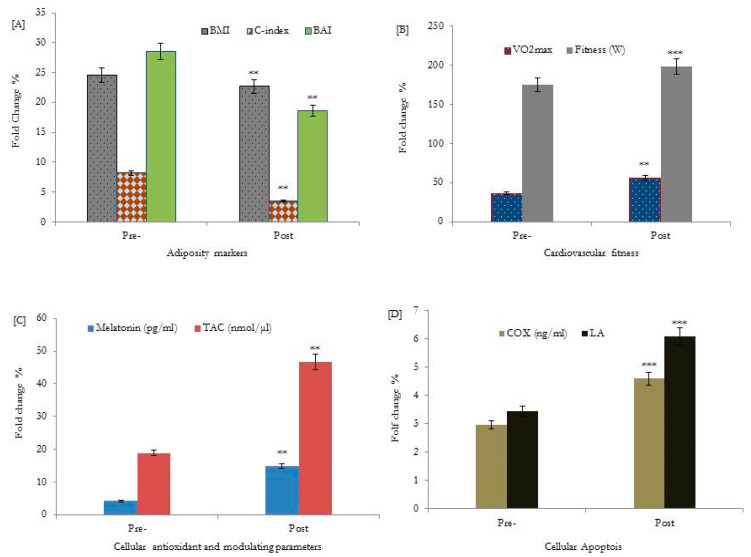
Effects of HIIT for 12 weeks on adiposity markers [**A**], cardiovascular fitness [**B**], cellular antioxidants and the melatonin modulating hormone [**C**], and cellular lymphocyte apoptosis [**D**] of healthy older adults. BMI: body mass index; WHtR: waist-to-height ratio; TAC: total antioxidant capacity; C-index: conicity index; BAI: body adiposity index; COX: cytochrome c oxidase (ng/mL). ** *p* < 0.01, bassline data of adiposity markers, VO_2_max, TAC, and cellular melatonin secretions were statistically significant compared to respective post-HIIT training values. *** *p* < 0.001, bassline data of fitness (W), and cellular apoptotic parameters; COX and LA % were statistically significant compared to respective values post-HIIT training intervention.

The results showed that adiposity markers, BMI, WHtR, CI-index, and BAI, were significantly decreased (*p* < 0.001) in older adults following HIIT exercise interventions for 12 weeks, as shown in Figure 2A and Table 2. In addition, there was an improvement in fitness scores (W; 196.5 ± 4.6, VO_2_max; 58.9 ± 2.5, *p* < 0.001) and glycemic control parameters (*p* < 0.01); FG, HbA1c (%), FI, and serum C-peptide were significantly improved following HIIT interventions, as shown in Figure 2B and Table 2.

Moreover, the melatonin hormone, TAC, cytochrome c oxidase COX, and lymphocyte apoptosis (LA) were studied as biological aging biomarkers. The data obtained showed that serum levels of the melatonin hormone (11.2 ± 2.3, *p* < 0.001), TAC (48.7 ± 7.1, *p* < 0.002), COX (3.7 ± 0.75, *p* < 0.001), and a higher percentage of lymphocyte apoptosis (5.2 ± 0.31, *p* < 0.003) were significantly increased following HIIT interventions, as shown in Figure 2C,D and Table 2.

Following 12 weeks of the HIIT-exercise program, the exercise duration showed a significant correlation with a higher level of serum melatonin (*p* = 0.001, r = 0.52), and the highest percentage of lymphocyte apoptosis (*p* = 0.001, r = 0.78)) (Table 3). Furthermore, melatonin and lymphocyte apoptosis expression showed a significant correlation with adiposity markers: BMI, WHtR, C-index, BAI, fitness, cytochrome c, and glycemic control variables, as shown in Figure 2A and Table 3. Additionally, lymphocyte apoptosis showed significant associations with the elevation of the melatonin hormone during the progression of the exercise training program (*p* = 0.002, r = 0.37), as shown in Figure 2D and Table 2.

## 4. Discussions

Our study indicated that participating in the HIIT-exercise training program for 12 weeks significantly increases physical performance and fitness, with a significant increase in serum melatonin level as an anti-aging hormone. Furthermore, HIIT-trained subjects showed a significant increase in the levels of TAC, % of lymphocytes apoptosis, and the levels of cytochrome c, and showed significant improvements in adiposity markers, BMI, WHtR, C-index, BAI, and glycemic control variables, especially FG, HbA1c (%), FI, and serum C-peptide. In addition, both melatonin levels and lymphocyte apoptosis as antiaging and physical performance cellular markers showed a significant correlation with exercise duration, fitness, TAC, and a reduction in adiposity markers and glycemic control parameters.

In our study, it seemed that HIIT exercise training for 12 weeks significantly improved adiposity markers (BMI, WHtR, C-index, BAI) and glycemic control variables, especially FG, HbA1c (%), FI, and serum C-peptide.

This means that HIIT may be an effective exercise strategy as an antiadiposity and antidiabetic agent to prevent potential metabolic health impairments among middle-aged people. Several recent studies reported that the interactions between different exercise training modes significantly improve aspects of cardiometabolic health, inflammation, and antioxidant status in inactive, overweight, and obese adults [49,50,51,52,53,54,55,56,57,58,59,60]. It was reported that combination treatments with aerobic and resistance training appear to be more beneficial for improving adiponectin, fasting insulin, fasting blood glucose, insulin resistance index, and triglyceride levels, compared to other exercise modalities [60]. In addition, similar to our results, the effectiveness of aerobic exercise (AE), resistance training (RT), combined aerobic and resistance training (CT), and high intensity interval training (HIIT) on body composition and inflammatory cytokine levels in overweight and obese individuals was recently reviewed using network meta-analysis (NMA). The findings of that review showed that HIIT, along with other combined modes of exercise, could effectively reduce the weight of overweight and obese patients [61,62].

Exercise training with different intensities showed significant changes in the hormonal section of endocrine glands, which play a role in strengthening the immune system and mobilizing lymphocyte cells [6]. Consistent with our data, previous research studies showed a significant increase in daytime melatonin levels in subjects following exercise training [63,64].

The exercise was shown to regulate melatonin secretion via a neural control mechanism, which occurs in controlled consecutive steps: First, there is an increase in the sympathetic discharge, then there is a release of noradrenalin, and finally, it elevates tryptophan levels in pineolocytes. Tryptophan was regarded as the precursor of melatonin synthesis, and its increase in blood circulation increases melatonin synthesis [65,66].

Furthermore, in HIIT-exercise-trained subjects, melatonin secretion significantly correlated with the duration of exercise and fitness scores. It proved that a longer exercise duration might improve melatonin secretion and overall physical performance. These results significantly matched those who reported increased melatonin with longer exercise activity [67]. However, a randomized controlled trial (RCT) showed that a 12-month moderate-intensity exercise intervention did not affect levels of aMT6s in men and women who were previously sedentary. In that study, moderate exercise training did not affect the levels of a urinary metabolite of melatonin 6-sulphatoxymelatonin (aMT6s) in subjects with previously sedentary activities [68].

HIIT and lower IIT exercise programs have effectively increased aerobic and anaerobic capacity in healthy, active men, which may induce metabolic and performance adaptations comparable to traditional endurance training [69]. In addition, HIIT exercise modes may promote an increase in melatonin synthesis and secretion [39,70] via modulating the relationships between melatonin and other hormones such as norepinephrine and epinephrine [39,70].

In this study, HIIT-exercise-trained subjects also showed significant improvements in the levels of total antioxidant capacity (TAC), adiposity markers BMI and WHtR, as well as glycemic control variables FG, HbA1c (%), FI, and serum C-peptide. Previous research showed that modulation and control of significant physiological processes, particularly DNA damage, oxidative stress, and hormonal changes in various cell types, depend mainly on the type of exercise, and its intensity, frequency, and duration as the training endpoint [68,69,70,71]. Furthermore, prolonged exercise training of at least moderate intensity improves TAC, insulin sensitivity, and HbA1c (%) levels, with improved adiposity markers in men with type 2 diabetes [72,73,74,75,76].

In addition, melatonin secretion in subjects showed a significant association with improved TAC, diabetes variables, and adiposity among subjects who participated in 12 weeks of HIIT-exercise training. Several experimental studies showed that supplementation of melatonin plays a significant role in suppressing body weight, visceral adiposity, plasma leptin, and insulin levels [77,78,79,80]. Thus, our data showed that HIIT exercise significantly improves the physical performance of healthy subjects by increasing melatonin expression, which may have potential therapeutic effects against insulin resistance, oxidative stress, and some aging-associated behavioral changes, and that the reduction in melatonin with aging alters metabolism and physical activity, resulting in obesity and associated detrimental metabolic consequences [81].

Physical exercise performance was increased in terms of both aerobic and anaerobic exercise capacities in subjects following high-intensity intermittent exercise (HIIE) training [80,82,83]. HIIT training increased muscle glycolytic and oxidative activities, particularly hexokinase, hydroxyl acyl-CoA dehydrogenase, citrate synthase, the expression of glucose transporter 4 (GLUT4), and peroxisome activated receptors, which collectively contain the master regulator of mitochondrial biogenesis in the skeletal muscles [84,85,86].

This study estimated TAC and COX as markers of physical performance. The data showed a significant increase in TAC and COX levels following HIIT training for 12 weeks. The data agreed with those who reported increased COX biogenesis in mice following moderate exercise of 60 min, 6 days a week [87]. Additionally, subjects with moderate to active physical activity showed a significant increase in TAC, and lower physical activity was associated with poor muscle biogenesis and lower muscle performance [88,89].

This study also showed that HITT- exercise for 12 weeks significantly increased lymphocyte apoptosis in healthy subjects. The data were significantly correlated with exercise duration, higher TAC and melatonin secretions, and a reduction or improvement of both adiposity and diabetes. The data obtained were in line with a previously estimated percentage of apoptotic lymphocytes isolated from the peripheral blood of normal subjects [90]. Furthermore, it was found previously that an exercise intensity threshold between 40 and 60% VO_2_ max significantly increases lymphocyte apoptosis [91].

The exercise showed efficiency in mobilizing and removing senescent T lymphocytes via apoptotic mechanisms from the bloodstream of healthy subjects following exercise training [92]. Apoptosis induced due to exercise may be caused by the interaction of death ligands and receptors such as the Fas receptor (CD95) and Fas ligand (CD95L), which were both significantly reported to increase following treadmill exercise tests [93]. The mobilized T lymphocytes from the peripheral lymphoid compartments were proposed to have a more advanced stage of biological aging with a reduction in capacity for clonal expansion than blood-resident T-cells, which ultimately were more sensitive to apoptosis than other blood lymphocytes [94].

Additionally, in this current study, there was a significant correlation between lymphocyte apoptosis and the duration of HIIT exercise training, which was significantly related to an increase in the level of melatonin with a prolonged time of daily exercise. This association supports the mechanistic role of melatonin in promoting lymphocyte apoptosis. Our data were in line with those who reported a significant role of melatonin in the stimulation of peripheral blood lymphocyte apoptosis, either via up-regulation of TNF, the major extrinsic mediator of apoptosis [94,95,96], or inhibition of lymphocyte division in the thymus and lymph nodes [96].

Our study had several limitations. Although our study generally showed the importance of HIIT exercise in enhancing physical performance among older adults and the significant association of lymphocyte apoptosis and melatonin as biological aging markers for aging changes among sedentary men aged 18–65 years, the lack of both a control group as well as long-term follow-up leads to a difficulty in seeing long-lasting changes in lymphocyte apoptosis and melatonin as a marker of biological aging. The lack of a control group is a major weakness. Our results can be interpreted as preliminary findings. Thus, further studies based on long follow-ups are recommended to understand the potential association of these biological parameters with phenotypic aging and to establish a future model of biological aging.

## 5. Conclusions

The main findings of this study were that HIIT exercise training interventions for 12 weeks significantly improved the adiposity markers, glycemic control parameters, and physical performance of sedentary, older adult men. In addition, melatonin secretion, % of lymphocyte apoptosis, COX activities, and TAC as biological aging markers were significantly increased following HIIT exercise training interventions for 12 weeks. The use of HIIT exercise seemed to be effective in improving biological aging, which will be adequate to support chronological age, especially regarding aging problems. However, subsequent studies are required with long-term follow up to consider HIIT as modulators for several cardiometabolic health problems in older individuals with obesity.

## Figures and Tables

**Table 1:** Demographic and clinical characteristics of the participants (n = 80).

Parameters	Subjects (N = 80)
Mean age (years)	42.5 ± 3.1
BMI (kg/m^2^)	24.6 ± 2.3
Waist (cm)	92 ± 1.2
Hips (cm)	105 ± 0.65
WHR	0.87 ± 0.54
C-index	8.45 ± 0.24
BAI	29.1 ± 3.7
Fasting glucose (FG; mg/dL)	85.9 ± 7.3
Serum C-peptide (ng/mL)	3.95 ± 1.7
HbA1c (%)	4.7 ± 0.25
Fasting insulin (FI; μU/mL)	23.3 ± 3.1

Data expressed as mean ± SD, BMI: body mass index; WHR: waist to hip ratio, C-index: Conicity index; BAI: Body Adiposity Index; HbA1c (%):glycated hemoglobin.

**Table 2:** Changes in glycemic markers, BMI, WHtR, fitness scores, serum melatonin, cytochrome c, TAC, and lymphocyte apoptosis (%) in healthy subjects following 12 weeks of the HIIT exercise program.

Parameters	Subjects (n = 80)		
	Pre-	Post-	*p*-Value
BMI (kg/m^2^)	24.6 ± 2.3	22.7 ± 1.9 *	0.001
WHtR	0.87 ± 0.54	0.84 ± 0.58 *	0.001
C-index	8.45 ± 0.24	3.48 ± 0.12 **	0.001
BAI	29.1 ± 3.7	17.9 ± 2.7 **	0.001
Fitness (W)	178.4 ± 2.8	196.5 ± 4.6 **	0.001
VO_2_max (mL/kg/min)	48.2 ± 1.3	58.9 ± 2.5 **	0.001
Fasting glucose (mg/dL)	85.9 ± 7.3	82.3 ± 5.1 *	0.01
Serum C-peptide (ng/mL)	3.95 ± 1.7	4.1 ± 1.2 **	0.01
HbA1c (%)	4.7 ± 0.25	4.3 ± 0.65 **	0.01
Fasting insulin (FI; μU/mL)	23.3 ± 3.1	28.1 ± 2.7 **	0.001
Serum melatonin (pg/mL)	3.18 ± 0.98	11.2 ± 2.3 **	0.001
Cytochrome c oxidase (ng/mL)	2.8 ± 0.86	3.7 ± 0.75 **	0.001
TAC (nmol/μL)	21.5 ± 3.1	48.7 ± 7.1 **	0.002
% of Lymphocyte Apoptosis	3.15 ± 0.47	5.2 ± 0.31 **	0.003

Data expressed as mean ± SD, percentage (%), * *p* < 0.01. ** *p* < 0.001 (pre vs. post) level values of subjects pre- and post-exercise training. BMI: body mass index, WHtR: waist-to-height ratio, C-index: conicity index; BAI: body adiposity index; TAC: total antioxidant capacity.

**Table 3:** Correlation coefficients analysis of serum melatonin and lymphocyte apoptosis (%) as an antiaging hormone with adiposity markers, fitness, TAC, and cytochrome C in healthy subjects following 12 weeks of the HIIT- exercise program.

	Serum Melatonin				Lymphocyte Apoptosis (%)			
	β	r	OR (95% CI)	*p*-Value	β	r	OR (95% CI)	*p*-Value
**BMI** (kg/m^2^)	0.12	0.15	1.9 (1.3–2.6)	0.01	0.34	0.87	2.7 (1.5–3.1)	0.11
**WHtR**	0.21	0.31	2.4 (1.8–3.1)	0.01	0.25	0.36	1.2 (0.96–1.8)	0.14
**C-index**	0.24	0.48	1.9 (1.5–3.2)	0.01	0.31	0.47	1.5 (0.96–2.3)	0.05
**BAI**	0.19	0.21	3.1 (1.8–5.7)	0.001	0.27	0.41	1.7 (0.96–2.6)	0.01
**Fitness (W)**	0.54	0.23	4.5 (3.2–6.7)	0.001	0.15	0.87	10.2 (5.6–14.8)	0.001
**TAC** (nmol/µL)	0.45	0.68	6.4 (4.8–11.4)	0.001	0.45	0.89	5.1 (3.5–7.3)	0.001
**HbA1c (%)**	0.32	0.28	2.7 (1.8–3.8)	0.002	0.43	0.941	3.7 (2.1–4.8)	0.001
**Cytochrome C** (COX) (ng/mL)	0.76	0.47	5.1 (2.8–4.3)	0.001	0.381	0.518	7.9 (4.7–10.9)	0.001
**Exercise duration**	0.15	0.52	2.8 (1.5–3.8)	0.001	0.75	0.78	8.6 (5.1–10.4)	0.001
**Serum melatonin** (pg/mL)	-	-	-	-	0.48	0.37	2.9 (1.5–4.3)	0.002

Multinomial logistic regressions were used to estimate correlations of the studied variables. BMI: body mass index; WHtR: waist-to-height ratio, TAC: total antioxidant capacity; C-index: conicity index; BAI: body adiposity index; beta regression coefficient (β); correlation coefficient (r); ORs: odds ratios; CI: confidence interval.

## Data Availability

The dataset supporting the study findings has been presented in this study and will be available from the corresponding authors upon a reasonable request.

## References

[1] Vos T., Flaxman A.D., Naghavi M., Lozano R., Michaud C., Ezzati M., Shibuya K., Salomon J.A., Abdalla S., Aboyans V. (2012). Years lived with disability (YLDs) for 1160 sequelae of 289 diseases and injuries 1990–2010: A systematic analysis for the Global Burden of Disease Study 2010. Lancet.

[2] Crimmins E.M. (2015). Lifespan and healthspan: Past, present, and promise. Gerontologist.

[3] Kaeberlein M. (2013). Longevity and aging. F1000Prime Rep..

[4] Kirkwood T.B.L. (2005). Understanding the odd science of aging. Cell.

[5] Gladyshev V.N. (2016). Aging: Progressive decline in fitness due to the rising deleteriome adjusted by genetic, environmental, and stochastic processes. Aging Cell.

[6] Kennedy B.K., Berger S.L., Brunet A., Campisi J., Cuervo A.M., Epel E.S., Franceschi C., Lithgow G.J., Morimoto R.I., Pessin J.E. (2014). Geroscience: Linking aging to chronic disease. Cell.

[7] Kaeberlein M., Rabinovitch P.S., Martin G.M. (2015). Healthy aging: The ultimate preventative medicine. Science.

[8] Ferrucci L., Fabbri E. (2018). Inflammageing: Chronic inflammation in ageing, cardiovascular disease, and frailty. Nat. Rev. Cardiol..

[9] Melzer D., Pilling L.C., Ferrucci L. (2020). The genetics of human ageing. Nat. Rev. Genet..

[10] Parker D.C., Bartlett B.N., Cohen H.J., Fillenbaum G., Huebner J.L., Kraus V.B., Pieper C., Belsky D.W. (2019). Association of Blood Chemistry Quantifications of Biological Aging With Disability and Mortality in Older Adults. J. Gerontol. A. Biol. Sci. Med. Sci..

[11] Belsky D.W., Moffitt T.E., Cohen A.A., Corcoran D.L., Levine M.E., Prinz J.A., Schaefer J., Sugden K., Williams B., Poulton R. (2018). Eleven Telomere, Epigenetic Clock, and Biomarker-Composite Quantifications of Biological Aging: Do They Measure the Same Thing?. Am. J. Epidemiol..

[12] Sebastiani P., Thyagarajan B., Sun F., Schupf N., Newman A.B., Montano M., Perls T.T. (2017). Biomarker signatures of aging. Aging Cell.

[13] Kuo P.-L., Schrack J.A., Levine M.E., Shardell M.D., Simonsick E.M., Chia C.W., Moore A.Z., Tanaka T., An Y., Karikkineth A. (2022). Longitudinal phenotypic aging metrics in the Baltimore Longitudinal Study of Aging. Nat. Aging.

[14] Elliott M.L., Caspi A., Houts R.M., Ambler A., Broadbent J.M., Hancox R.J., Harrington H., Hogan S., Keenan R., Knodt A. (2021). Disparities in the pace of biological aging among midlife adults of the same chronological age have implications for future frailty risk and policy. Nat. Aging.

[15] Kuo P., Schrack J.A., Shardell M.D., Levine M., Moore A.Z., An Y., Elango P., Karikkineth A., Tanaka T., Cabo R. (2020). A roadmap to build a phenotypic metric of ageing: Insights from the Baltimore Longitudinal Study of Aging. J. Intern. Med..

[16] Ferrucci L., Cooper R., Shardell M., Simonsick E.M., Schrack J.A., Kuh D. (2016). Age-related change in mobility: Perspectives from life course epidemiology and geroscience. J. Gerontol. A Biol. Sci. Med. Sci..

[17] Horvath S., Raj K. (2018). DNA methylation-based biomarkers and the epigenetic clock theory of ageing. Nat. Rev. Genet..

[18] Allen P.D., Bustin S.A., Newland A.C. (1993). The role of apoptosis (programmed cell death) in haemopoiesis and the immune system. Blood Rev..

[19] Granville D.J., Carthy C.M., Hunt D.W., McManus B.M. (1998). Apoptosis: Molecular aspects of cell death and disease. Lab. Investig..

[20] Berridge M.J. (1997). Lymphocyte activation in health and disease. Crit. Rev. Immunol..

[21] Genestier L., Bonnefoy-Berard N., Revillard J.P. (1999). Apoptosis of activated peripheral T cells. Transpl. Proc..

[22] Schuster C., Gauer F., Malan A., Recio J., Pevet P., Masson-Pevet M. (2001). The circadian clock, light/dark cycle and melatonin are differentially involved in the expression of daily and photoperiodic variations in mtl melatonin receptors in Siberian and Syrian hamsters. Neuroendocrinology.

[23] Oxenkrug G., Requintina P., Bachurin S. (2001). Antioxidant and antiaging activity of N-acetylserotonin and melatonin in the in vivo models. Ann. N. Y. Acad. Sci..

[24] Li F., Li S., Li H.B., Deng G.F., Ling W.H., Wu S., Xu X.R., Chen F. (2013). Antiproliferative activity of peels, pulps and seeds of 61 fruits. J. Funct. Foods.

[25] Galano A., Tan D.X., Reiter R.J. (2013). On the free radical scavenging activities of melatonin’s metabolites, AFMK and AMK. J. Pineal Res..

[26] Galano A., Tan D.X., Reiter R.J. (2012). Melatonin as a natural ally against oxidative stress: A physicochemical examination. J. Pineal Res..

[27] Pandi-Perumal S.R., Zisapel N., Srinivasan V., Cardinali D.P. (2005). Melatonin and sleep in aging population. Exp. Gerontol..

[28] Agil A., Elmahallawy E.K., Rodriguez-Ferrer J.M., Adem A., Bastaki S.M., Al-Abbadi I., Fino Solano Y.A., Navarro-Alarcon M. (2015). Melatonin increases intracellular calcium in the liver, muscle, white adipose tissuesand pancreas of diabetic obese rats. Food Funct..

[29] Agil A., El-Hammadi M., Jimenez-Aranda A., Tassi M., Abdo W., Fernandez-Vazquez G., Reiter R.J. (2015). Melatonin reduces hepatic mitochondrial dysfunction in diabetic obese rats. J. Pineal Res..

[30] Valenzuela P.L., Castillo-García A., Morales J.S., de la Villa P., Hampel H., Emanuele E., Lista S., Lucia A. (2020). Exercise benefits on Alzheimer’s disease: State-of-the-science. Ageing Res. Rev..

[31] AlAnazi A., Alghadir A.H., Gabr S.A. (2022). Handgrip Strength Exercises Modulate Shoulder Pain, Function, and Strength of Rotator Cuff Muscles of Patients with Primary Subacromial Impingement Syndrome. Biomed Res. Int..

[32] Alghadir A.H., Gabr S.A. (2020). Hormonal Function Responses to Moderate Aerobic Exercise in Older Adults with Depression. Clin. Interv. Aging.

[33] Alghadir A.H., Gabr S.A., Al-Momani M., Al-Momani F. (2021). Moderate aerobic training modulates cytokines and cortisol profiles in older adults with cognitive abilities. Cytokine.

[34] Mota M., Pănuş C., Mota E., Lichiardopol C., Vladu D., Toma E. (2004). the metabolic syndrome—A multifaced disease. Rom. J. Intern. Med..

[35] Mackinnon L.T. (1994). current challenges and future expectations in exercise immunology: Back to the future. Med. Sci. Sports Exerc..

[36] Venkatraman J.T., Fernandes G. (1997). Exercise, immunity and aging. Aging.

[37] Viswanathan A.N., Schernhammer E.S. (2009). Circulating melatonin and the risk of breast and endometrial cancer in women. Cancer Lett..

[38] Escames G., Ozturk G., Bano-Otalora B., Pozo M.J., Madrid J.A., Reiter R.J., Serrano E., Concepción M., Acuña-Castroviejo D. (2012). Exercise and melatonin in humans: Reciprocal benefits. J. Pineal Res..

[39] Mastaloudis A., Leonard S.W., Traber M.G. (2001). Oxidative stress in athletes during extreme endurance exercise. Free Radic. Biol. Med..

[40] Hood D., Uguccioni G., Vainshtein A., D’souza D. (2011). Mechanisms of exercise-induced mitochondrial biogenesis in skeletal muscle: Implications for health and disease. Compr. Physiol..

[41] Karstoft K., Winding K., Knudsen S.H., James N.G., Scheel M.M., Olesen J., Holst J.J., Pedersen B.K., Solomon T.P.J. (2014). Mechanisms behind the superior effects of interval vs continuous training on glycaemic control in individuals with type 2 diabetes: A randomised controlled trial. Diabetologia.

[42] Youssef L., Granet J., Marcangeli V., Dulac M., Hajj-Boutros G., Reynaud O., Buckinx F., Gaudreau P., Morais J.A., Mauriège P. (2022). Clinical and Biological Adaptations in Obese Older Adults Following 12-Weeks of High-Intensity Interval Training or Moderate-Intensity Continuous Training. Healthcare.

[43] Trachsel L.D., David L.P., Gayda M., Henri C., Hayami D., Thorin-Trescases N., Thorin E., Blain M.A., Cossette M., Lalongé J. (2019). The impact of high-intensity interval training on ventricular remodeling in patients with a recent acute myocardial infarction-A randomized training intervention pilot study. Clin. Cardiol..

[44] Kercher V.M.M., Kercher K., Levy P., Bennion T., Alexander C., Amaral P.C., Batrakoulis A., Chávez L.F.J.G., Cortés-Almanzar P., Haro J.L. (2023). 2023 Fitness Trends from Around the Globe. ACSM’s Health Fit. J..

[45] Aekplakorn W., Kosulwat V., Suriyawongpaisal P. (2006). Obesity indices and cardiovascular risk factors in Thai adults. Int. J. Obes..

[46] Bergman R.N., Stefanovski D., Buchanan T.A., Sumner A.E., Reynolds J.C., Sebring N.G., Xiang A.H., Watanabe R.M. (2011). A better index of body adiposity. Obesity.

[47] Bull F.C., Maslin T.S., Armstrong T. (2009). Global physical activity questionnaire (GPAQ): Nine country reliability and validity study. J. Phys. Act. Health.

[48] Trinh O.T., Nguyen N.D., Van Der Ploeg H.P., Dibley M.J., Bauman A. (2009). Testretest repeatability and relative validity of the Global Physical Activity Questionnaire in a developing country context. J. Phys. Act. Health.

[49] Cochran A.J., Percival M.E., Tricarico S., Little J.P., Cermak N., Gillen J.B., Tarnopolsky M.A., Gibala M.J. (2014). Intermittent and continuous high-intensity exercise training induce similar acute but different chronic muscle adaptations. Exp. Physiol..

[50] Kwak L., Kremers S.P., Bergman P., Ruiz J.R., Rizzo N.S., Sjöström M. (2009). Associations between physical activity, fitness, and academic achievement. J. Pediatr..

[51] Boyum M. (1968). Isolation of mononuclear cells and granulocytes from human blood. Scand. J. Clin. Lab. Investig..

[52] Mendelsohn J., Skinner A., Kornfield S. (1971). The rapid induction by phytohemagglutinin of increased alpha-aminoisobutyric acid uptake by lymphocytes. J. Clin. Investig..

[53] Dolye A.l., Griffiths J.B. (2000). Haemocytometer cell count and viability studies. Cell and Tissue Culture for Medical Research.

[54] Vacca L.L. (1985). Acridine orange. Laboratory Manual of Histochemistry.

[55] Alghadir A.H., Gabr S.A., Iqbal Z.A., Al-Eisa E. (2019). Association of physical activity, vitamin E levels, and total antioxidant capacity with academic performance and executive functions of adolescents. BMC Pediatr..

[56] Alghadir A.H., Gabr S.A. (2016). Efficacy of Rhus coriaria (sumac) juice in reducing muscle pain during aerobic exercise. Physiol. Int..

[57] Gabr S.A., Gabr N.S., Elsaed W.M. (2019). Aqueous Green Tea Extract and Prediction of Fibrosis in Lipopolysaccharide Intoxicated Rats. Int. J. Pharmacol..

[58] Alghadir A.H., Gabr S.A., Al-Eisa E.S. (2016). Effects of Moderate Aerobic Exercise on Cognitive Abilities and Redox State Biomarkers in Older Adults. Oxidative Med. Cell. Longev..

[59] Batrakoulis A., Jamurtas A.Z., Draganidis D., Georgakouli K., Tsimeas P., Poulios A., Syrou N., Deli C.K., Papanikolaou K., Tournis S. (2021). Hybrid Neuromuscular Training Improves Cardiometabolic Health and Alters Redox Status in Inactive Overweight and Obese Women: A Randomized Controlled Trial. Antioxidants.

[60] Hejazi K., Wong A. (2023). Effects of exercise training on inflammatory and cardiometabolic health markers in overweight and obese adults: A systematic review and meta-analysis of randomized controlled trials. J. Sports Med. Phys. Fit..

[61] Jensen M.D., Ryan D.H., Apovian C.M., Ard J.D., Comuzzie A.G., Donato K.A., Hu F.B., Hubbard V.S., Jakicic J.M., Kushner R.F. (2014). 2013 Aha/Acc/Tos Guideline for the Management of Overweight and Obesity in Adults: A Report of the American College of Cardiology/American Heart Association Task Force on Practice Guidelines and the Obesity Society. Circulation.

[62] Wang S., Zhou H., Zhao C., He H. (2022). Effect of Exercise Training on Body Composition and Inflammatory Cytokine Levels in Overweight and Obese Individuals: A Systematic Review and Network Meta-Analysis. Front. Immunol..

[63] Skrinar G.S., Bullen B.A., Reppert S.M., Peachey S.E., Turnbull B.A., McArthur J.W. (1989). Melatonin response to exercise training in women. J. Pineal Res..

[64] Pilaczyńska-Szcześniak L., Karolkiewicz J., Strzelczyk A., Stankiewicz K., Osiński W., Stemplewski R., Szeklicki R. (2004). Melatonin concentrations and other parameters of blood antioxidant defense system in elderly men with various levels of physical activity. Pol. Arch. Intern. Med..

[65] Reiter R.J. (1991). Pineal melatonin: Cell biology of its synthesis and of its physiological interactions. Endocr. Rev..

[66] Blomstrand E., Celsing F., Newsholme E.A. (1998). Changes in plasma concentrations of aromatic and branched-chain amino acids during sustained exercise in man and their possible role in fatigue. Acta Physiol. Scand..

[67] Follenius M., Weibel L., Brandenberger G. (1995). Distinct modes of melatonin secretion in normal men. J. Pineal Res..

[68] Knight J.A., Thombson S., Raboud J.M., Hoffman B.R. (2005). Light and Exercise and Melatonin Production in Women. Am. J. Epidemiol..

[69] Thrift A.P., Xiao L., Patel S.R., Tworoger S.S., McTiernan A., Duggan C. (2014). Effects of physical activity on melatonin levels in previously sedentary men and women. Cancer Epidem. Biomar. Prev..

[70] Mougios V., Bahrke M.S. (2010). Exercisemetabolism. Exercise Biochemistry.

[71] Solberg P.A., Halvari H., Ommundsen Y., Hopkins W.G. (2014). A 1-year follow-up on effects of exercise programs on wellbeing in older adults. J. Aging Phys. Act..

[72] Aly F., Alghadir A., Gabr S. (2014). Adiponectin response to supervised aerobic training in type II diabetic patients. Asian Biomed..

[73] Yoshida H., Ishikawa T., Suto M., Kurosawa H., Hirowatari Y., Ito K., Yanai H., Tada N., Suzuki M. (2010). Effects of supervised aerobic exercise training on serum adiponectin and parameters of lipid and glucose metabolism in subjects with moderate dyslipidemia. J. Atheroscler. Thromb..

[74] Goodyear L.J., Kahn B.B. (1998). Exercise, glucose transport, and insulin sensitivity. Ann. Rev. Med..

[75] Sajad A., Amir H.H., Mohammad R.H. (2007). Effects of resistance versus endurance training on serum adiponectin and insulin resistance index. Eur. J. Endocrinol..

[76] Cipryan L. (2017). IL-6, Antioxidant Capacity and Muscle Damage Markers Following High-Intensity Interval Training Protocols. J. Hum. Kinet..

[77] Puchalski S.S., Green J.N., Rasmussen D.D. (2003). Melatonin effects on metabolism independent of gonad function. Endocrine.

[78] Bartness T.J., Demas G.E., Song C.K. (2002). Seasonal changes in adiposity: The roles of the photoperiod, melatonin and other hormones, and sympathetic nervous system. Exp. Biol. Med..

[79] Rasmussen D.D., Mitton D.R., Larsen S.A., Yellon S.M. (2001). Aging-dependent changes in the effect of daily melatonin supplementation on rat metabolic and behavioral responses. J. Pineal Res..

[80] Wolden-Hanson T., Mitton D.R., McCants R.L., Yellon S.M., Wilkinson C.W., Matsumoto A.M., Rasmussen D.D. (2000). Daily melatonin administration to middle-aged male rats suppresses body weight, intraabdominal adiposity, and plasma leptin and insulin independent of food intake and total body fat. Endocrinology.

[81] Tabata I., Irisawa K., Kouzaki M., Nishimura K., Ogita F., Miyachi M. (1997). Metabolic profile of high-intensity intermittent exercises. Med. Sci. Sport. Exerc..

[82] Tabata I., Nishimura K., Kouzaki M., Hirai Y., Ogita F., Miyachi M., Yamamoto K. (1996). Effects of moderate-intensity endurance and high-intensity intermittent training on anaerobic capacity and VO(2max). Med. Sci. Sports Exerc..

[83] Fujimoto E., Machida S., Higuchi M., Tabata I. (2010). Effects of nonexhaustive bouts of high-intensity intermittent swimming training on GLUT-4 expression in rat skeletal muscle. J. Physiol. Sci..

[84] Terada S., Kawanaka K., Goto M., Shimokawa T., Tabata I. (2005). Effects of high-intensity intermittent swimming on PGC protein expression in rat skeletal muscle. Acta Physiol. Scand..

[85] Terada S., Tabata I., Higuchi M. (2004). Effect of high-intensity intermittent swimming training on fatty acid oxidation enzyme activity in rat skeletal muscle. Jpn. J. Physiol..

[86] Terada S., Yokozeki T., Kawanaka K., Ogawa K., Higuchi M., Ezaki O., Tabata I. (2001). Effects of high-intensity swimming training on GLUT-4 and glucose transport activity in rat skeletal muscle. J Appl. Physiol..

[87] Sun Y., Qi Z., He Q., Cui D., Qian S., Ji L., Ding S. (2015). The effect of treadmill training and N-acetyl-l-cysteine intervention on biogenesis of cytochrome c oxidase (COX). Free. Radic. Biol. Med..

[88] Al-Eisa E.S., Alghadir A.H., Gabr S.A. (2016). Correlation between vitamin D levels and muscle fatigue risk factors based on physical activity in healthy older adults. Clin. Interv. Aging.

[89] Georgakouli K., Manthou E., Fatouros I.G., Georgoulias P., Deli C.K., Koutedakis Y., Theodorakis Y., Jamurtas A.Z. (2017). Enhanced erythrocyte antioxidant status following an 8-week aerobic exercise training program in heavy drinkers. Alcohol.

[90] Israa F., Al-Samaraee F., Al-Ani, Inaam A.R. (2002). Experimental model for lymphocyte apoptosis In Vitro. J. Fac. Med..

[91] Navalta J.W., Sedlock D.A., Park K.S. (2007). Effect of exercise intensity on exercise-induced lymphocyte apoptosis. Int. J. Sports Med..

[92] Mars M., Govender S., Weston A., Naicker V., Chuturgoon A. (1998). High intensity exercise: A cause of lymphocyte apoptosis?. Biochem. Biophys. Res. Comm..

[93] Curtin J.F., Cotter T.G. (2003). Live and let die: Regulatory mechanisms in Fas-mediated apoptosis. Cell. Signal..

[94] Simpson R.J., Florida-James G.D., Cosgrove C., Whyte G.P., Macrae S., Pircher H., Guy K. (2007). High-intensity exercise elicits the mobilization of senescent T lymphocytes into the peripheral blood compartment in human subjects. J. Appl. Physiol..

[95] Liu F., Ng T., Fung M. (2001). Pineal indoles stimulate the gene expression of immunomodulating cytokines. J. Neural Transm..

[96] Sáinz R.M., Mayo J.C., Kotler M., Uría H., Antolín I., Rodríguez C. (1998). Melatonin decreases mRNA for histone H4 in thymus of young rats. Life Sci..

